# Supplementary material for: Tailing and degradation of Argonaute-bound small RNAs protect the genome from uncontrolled RNAi
Source: Nat Commun. 2017 May 25;8:15332. doi: 10.1038/ncomms15332 (PMC5458512; doi:10.1038/ncomms15332)
Supplement: Supplementary Information — Supplementary figures, supplementary tables and supplementary references. [file ncomms15332-s1.pdf]

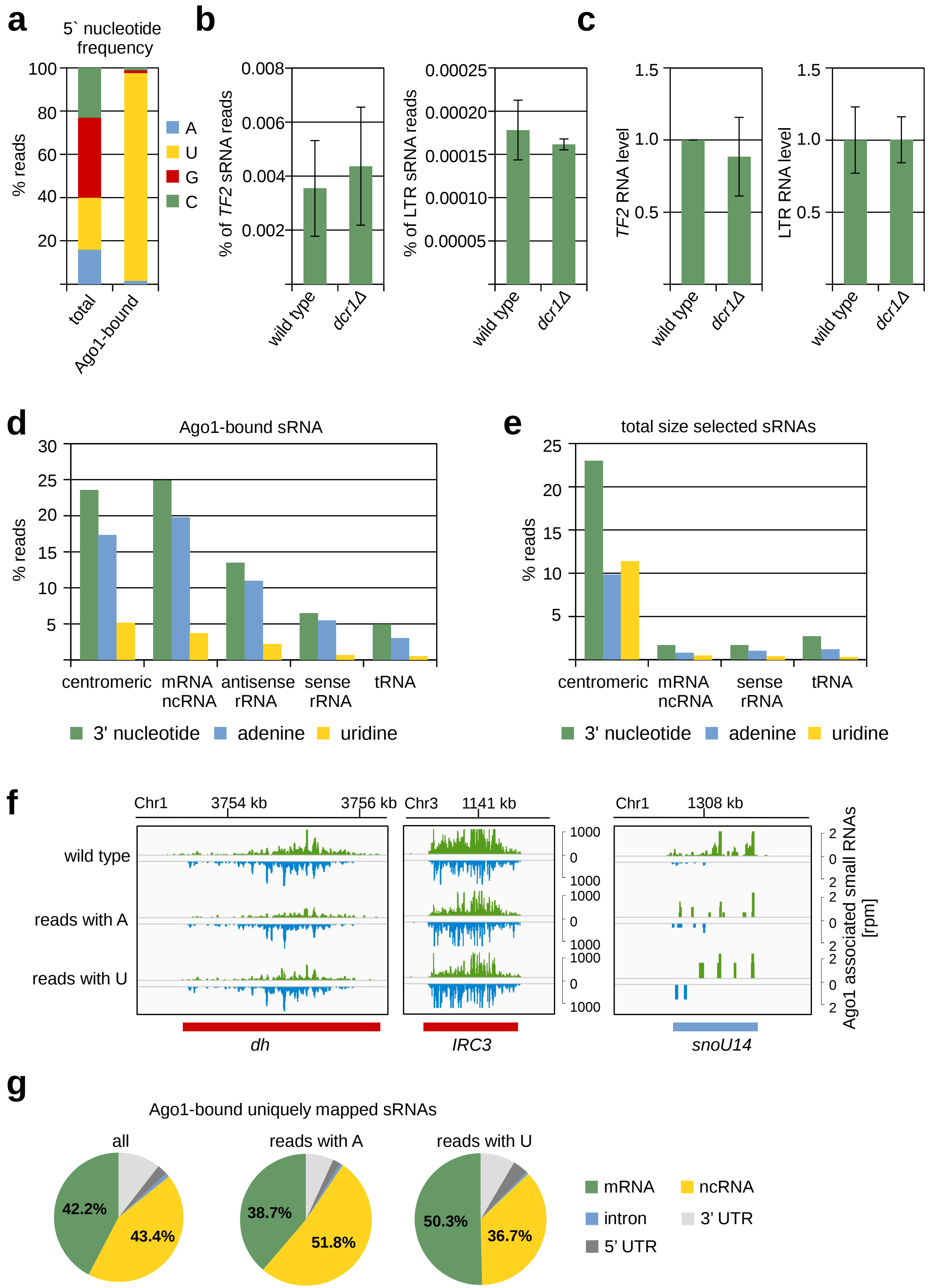

Supplementary Figure 1

Supplementary Figure 1. Argonaute-bound small RNAs have non-templated nucleotides at their 3' end.

(a) 5' nucleotide preference of Argonaute-bound and size selected total small RNAs in wild type cells. 5' U bias indicates Argonaute association.

(b) Quantification of Argonaute-bound small RNAs that map to TF2 or LTR elements in indicated strains. RNAi does not target transposable elements in fission yeast wild type cells. Error bars indicate s.e.m. of two independent small RNA sequencing experiments.

(c) Quantification of TF2 and LTR transcripts in indicated strains by RNA sequencing. Neither TF2 or LTR are up-regulated in RNAi mutants. Error bars indicate s.e.m. of two independent RNA sequencing experiments.

(d) Quantification of small RNAs that have non-templated nucleotides at the 3' end in different classes of Argonaute-bound small RNAs. Small RNAs that originate from centromeric region, mRNA and ncRNA and antisense rRNA are abundantly modified at the 3' end. Small RNAs originating from tRNAs and sense rRNA are modified less frequently.

(e) Quantification of small RNAs that have non-templated nucleotides at the 3' end in different classes of size selected fraction. Only small RNAs that originate from centromeric region are abundantly modified at the 3' end. Small RNAs originating from mRNA, ncRNA, tRNAs and rRNA are modified less frequently suggesting that these small RNAs are not loaded on Argonaute.

(f) Argonaute-bound small RNA reads from wild type cells were plotted over centromeric region and euchromatic gene. Reads having non-templated adenine(s) or uridine(s) at the 3' end are shown in separated tracks. The location of genes is indicated below the small RNA peaks. Reads from + and - strands are depicted in green and blue, respectively. Scale bars on the right denote small RNA reads numbers normalized per one million reads.

(g) Argonaute-bound small RNAs from wild type cells were analyzed by high-throughput sequencing and classified as indicated below the pie charts. Pie charts illustrate percentages for the individual small RNA classes relative to the total number of reads for each strain.

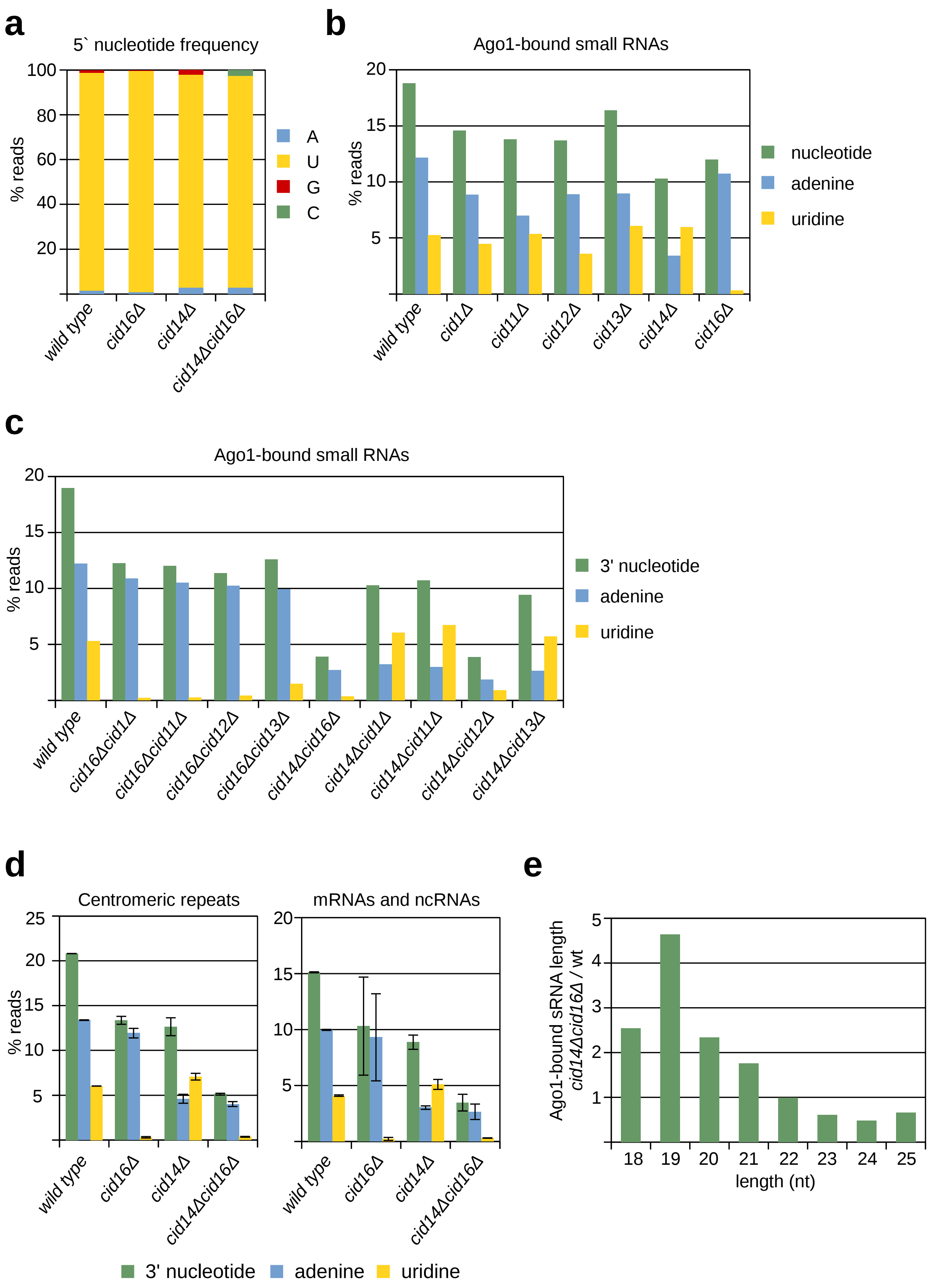

Supplementary Figure 2

Supplementary Figure 2. Cid14 adenylates and Cid16 urdinylates the 3' end of small RNAs

(a) 5' nucleotide preference of Argonaute-bound small RNAs in indicated cells. Strong preference for 5' U indicates Argonaute association**.**

(b) Quantification of Argonaute-bound small RNAs that have non-templated nucleotides at the 3' end in indicated cells. Deletion of Cid14 shows strong reduction in addition of non-templated adenine(s). Deletion of Cid16 shows strong reduction in addition of non-templated uridine(s).

(c) Quantification of Argonaute-bound small RNAs that have non-templated nucleotides at the 3' end in indicated cells. Deletion of Cid14 and Cid12 shows additional reduction in addition of non-templated nucleotides.

(d) Quantification of Argonaute-bound small RNAs from centromeric region and from mRNAs and ncRNAs that have non-templated nucleotides at the 3' end in indicated cells. Error bars indicate s.e.m. of two independent small RNA sequencing experiments.

(e) Length of Argonaute-bound small RNAs in *cid14Δcid16Δ* cells. Small RNAs in *cid14Δcid16Δ* cells are shorter than in wild type cells, consistent with lack of tailing.

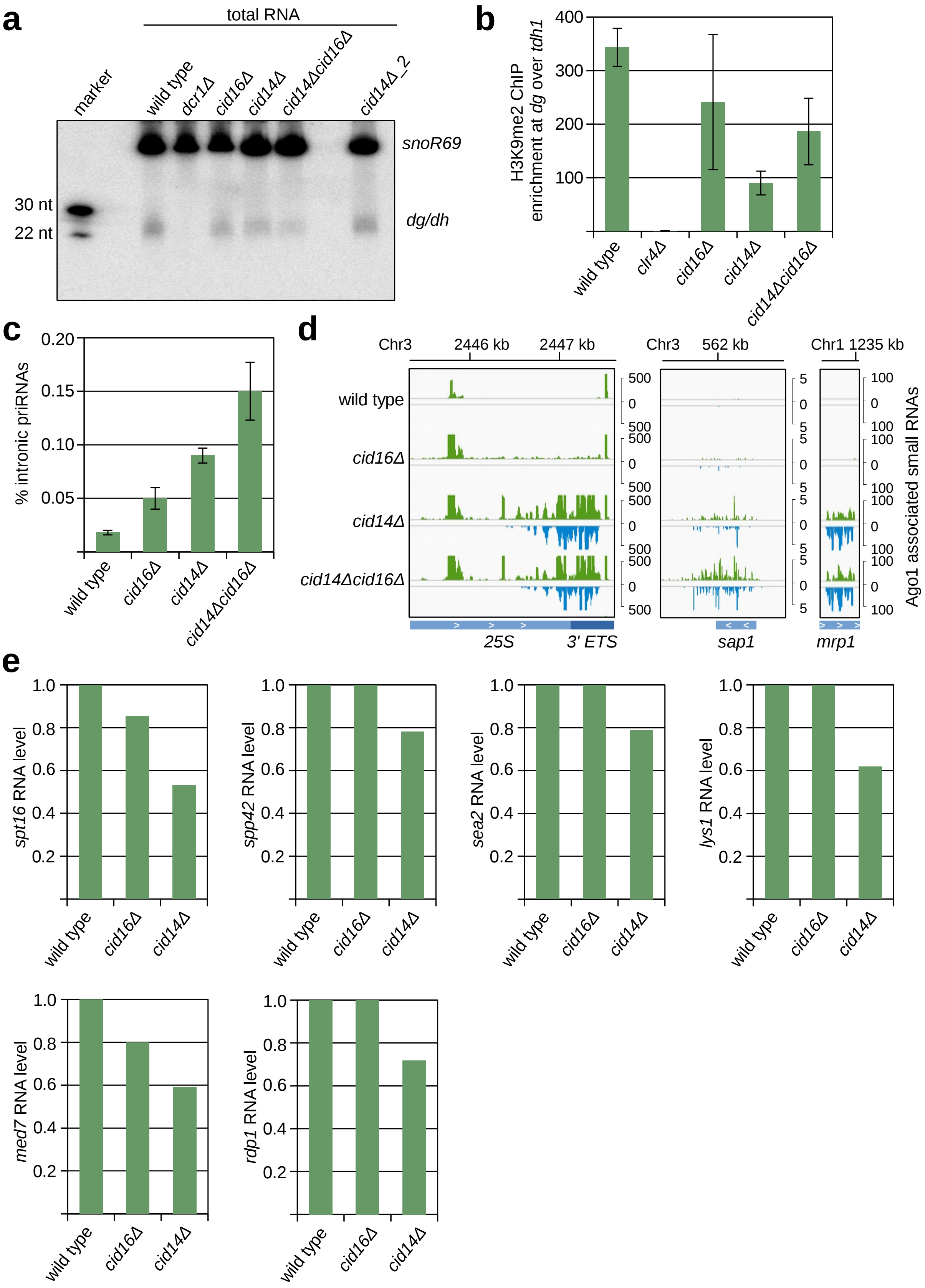

Supplementary Figure 3

Supplementary Figure 3. Accumulation of antisense priRNAs triggers spurious RNAi in *cid14Δ* cells

(a) Northern blotting showing *dg* and *dh* transcripts from total RNA isolated from the indicated strains. In *cid14Δcid16Δ* cells centromeric small RNAs are reduced. Two independent *cid14Δ* strains show near wild type levels of centromeric siRNAs. Strain *cid14Δ_2* is from Bühler *et al*, 2007[1].

(b) ChIP experiment showing that in *cid16Δ, cid14Δ* and *cid14cid16Δ* cells H3K9me2 is partially lost at centromeric *dg* repeats. Error bars indicate s.e.m. of three independent experiments.

(c) Quantification of intronic Argonaute-bound siRNAs and priRNAs in indicated strains. Intronic siRNAs and priRNA accumulate in *cid16Δ, cid14Δ* and *cid14cid16Δ* cells. Error bars indicate s.e.m. of two independent small RNA sequencing experiments.

(d) Argonaute-bound small RNA reads from indicated strains were plotted over euchromatic genes and rDNA. The location of genes is indicated below the small RNA peaks. Reads from + and - strands are depicted in green and blue, respectively. Scale bars on the right denote small RNA reads numbers normalized per one million reads. In *cid14Δ* and *cid14Δcid16Δ* cells, RNAi targets rDNA and many euchromatic genes.

(e) Read counts of RNA sequencing from indicated strains for genes shown in Fig. 3d and for *rdp1*.

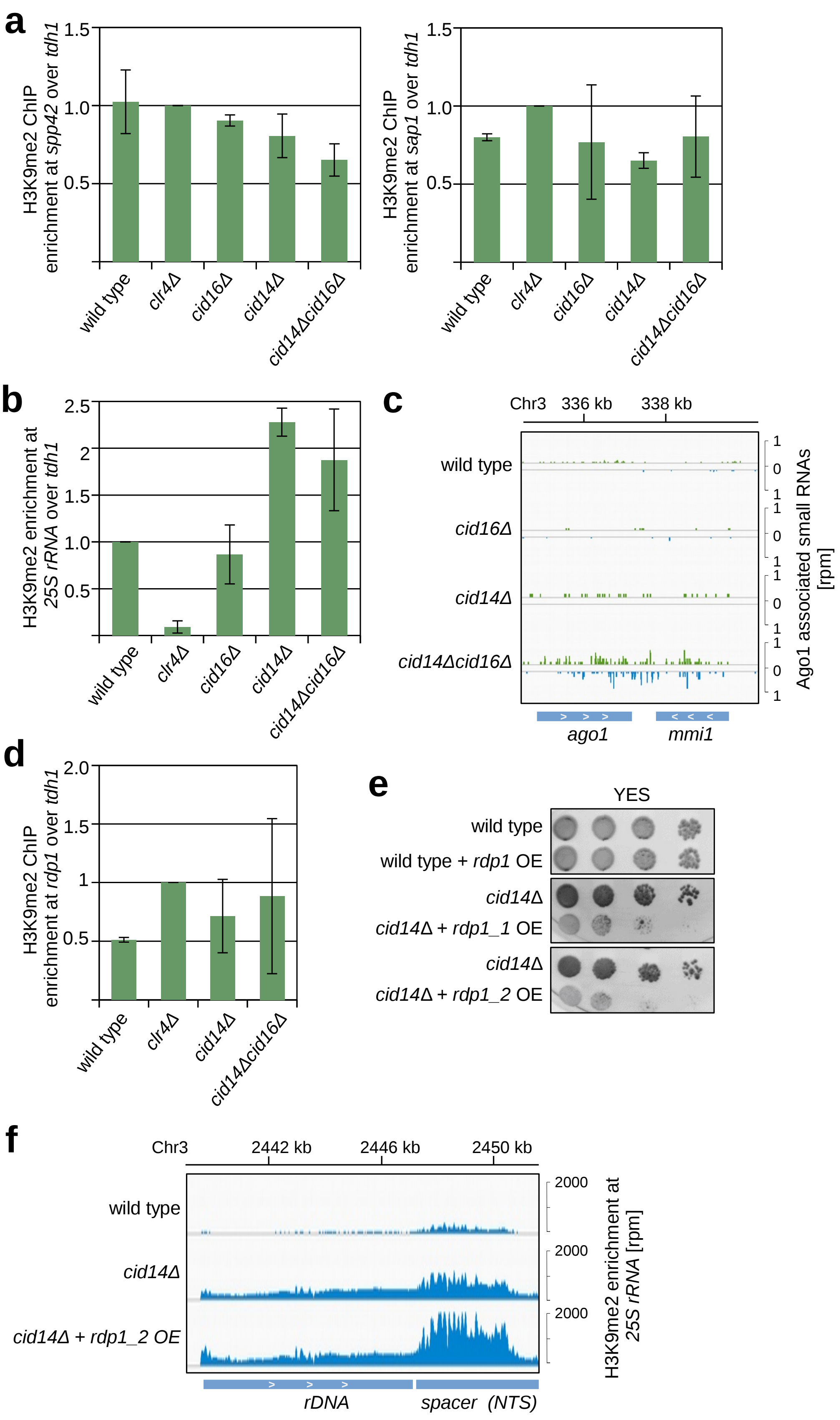

Supplementary Figure 4

Supplementary Figure 4. RNAi silences *rdp1* to reduce spurious RNAi

(a) ChIP experiment showing that in *cid16Δ, cid14Δ* and *cid14cid16Δ* cells H3K9me2 is not established at targeted genes indicating that silencing is heterochromatin independent. Error bars indicate s.e.m. of three independent experiments.

(b) ChIP experiment showing that in *cid14Δ* and *cid14cid16Δ* cells H3K9me2 is increased at rDNA. Error bars indicate s.e.m. of three independent experiments.

(c) Argonaute-bound small RNA reads from indicated strains were plotted over euchromatic genes. The location of genes is indicated below the small RNA peaks. Reads from + and - strands are depicted in green and blue, respectively. Scale bars on the right denote small RNA reads numbers normalized per one million reads. In *cid14Δcid16Δ* cells, RNAi targets *ago1*.

(d) ChIP experiment showing that in *cid16Δ, cid14Δ* and *cid14cid16Δ* cells H3K9me2 is not established at *rdp1* locus indicating that silencing of *rdp1* gene is heterochromatin independent. Error bars indicate s.e.m. of three independent experiments.

(e) Growth assay showing strong reduction in viability of *cid14Δ* cells that over-express *rdp1* gene. Cells were growing for three days before imaging.

(f) ChIPseq experiment showing that H3K9me2 is increased at rDNA in *cid14Δ* cells that over-express Rdp1.

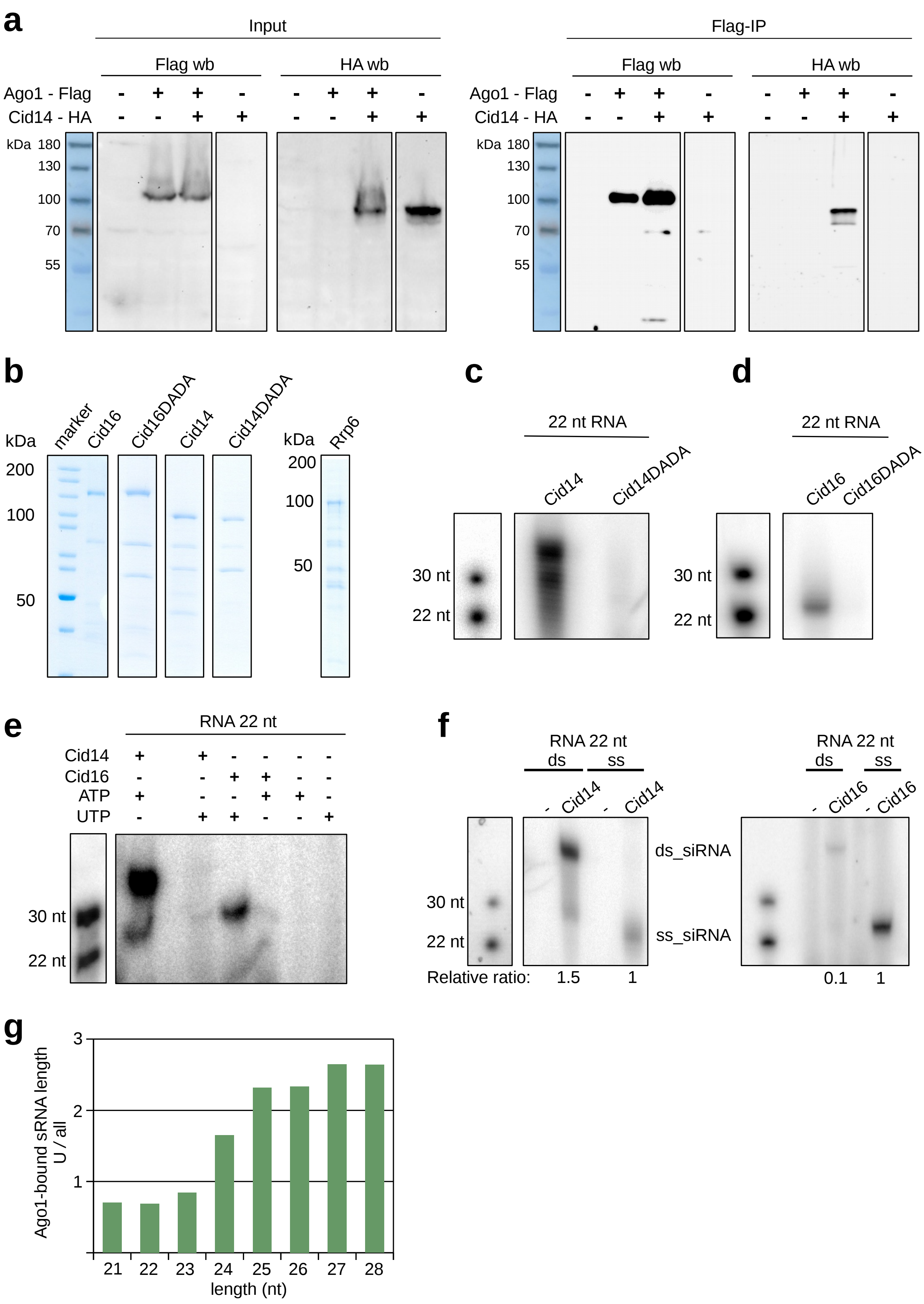

Supplementary Figure 5

Supplementary Figure 5. Cid14/Cid16 and Rrp6 degrade Argonaute-bound small RNAs

(a) Western blotting analysis of co-immunoprecipitation assay showing that Argonaute interacts with Cid14 in vivo.

(b) Coomassie stained SDS polyacrylamide gel showing purified Cid16, Cid16DADA, Cid14, Cid14DADA and Rrp6. Cid14, Cid14DADA and Rrp6 proteins were expressed and purified from *E. coli*. Cid16 and Cid16DADA were expressed and purified from *S. pombe.*

(c, d) Autoradiograph of denaturing polyacrylamide gel showing Cid14 and Cid16 activity on free small RNA. 22 nucleotide long small RNA was incubated with Cid14/Cid16 and α-^{32}P ATP/UTP and small RNA was analyzed on a polyacrylamide gel. Cid14 adds 10-20 adenines and Cid16 adds 1-2 uridine(s). Cid14DADA and Cid16DADA active site point mutants show no activity.

(e) Autoradiograph of denaturing polyacrylamide gel showing Cid14 and Cid16 activity on free small RNA and specificity for ATP and UTP, respectively. 22 nucleotide long small RNA was incubated with Cid14/Cid16 and α-^{32}P ATP/UTP and small RNA was analyzed on a polyacrylamide gel. Cid14 adds adenines and Cid16 adds uridine(s).

(f) Autoradiograph of polyacrylamide gel showing Cid14 and Cid16 activity on free dsRNA and ssRNA. 22 nucleotide single and double stranded siRNA were incubated with Cid14 (α-^{32}P ATP) and Cid16 (α-^{32}P UTP) and small RNA was analyzed on a polyacrylamide gel.

(g) Length of uridylated Argonaute-bound small RNAs in wild type cells. Uridylated small RNAs are longer than average Argonaute-bound small RNAs in wild type cells.

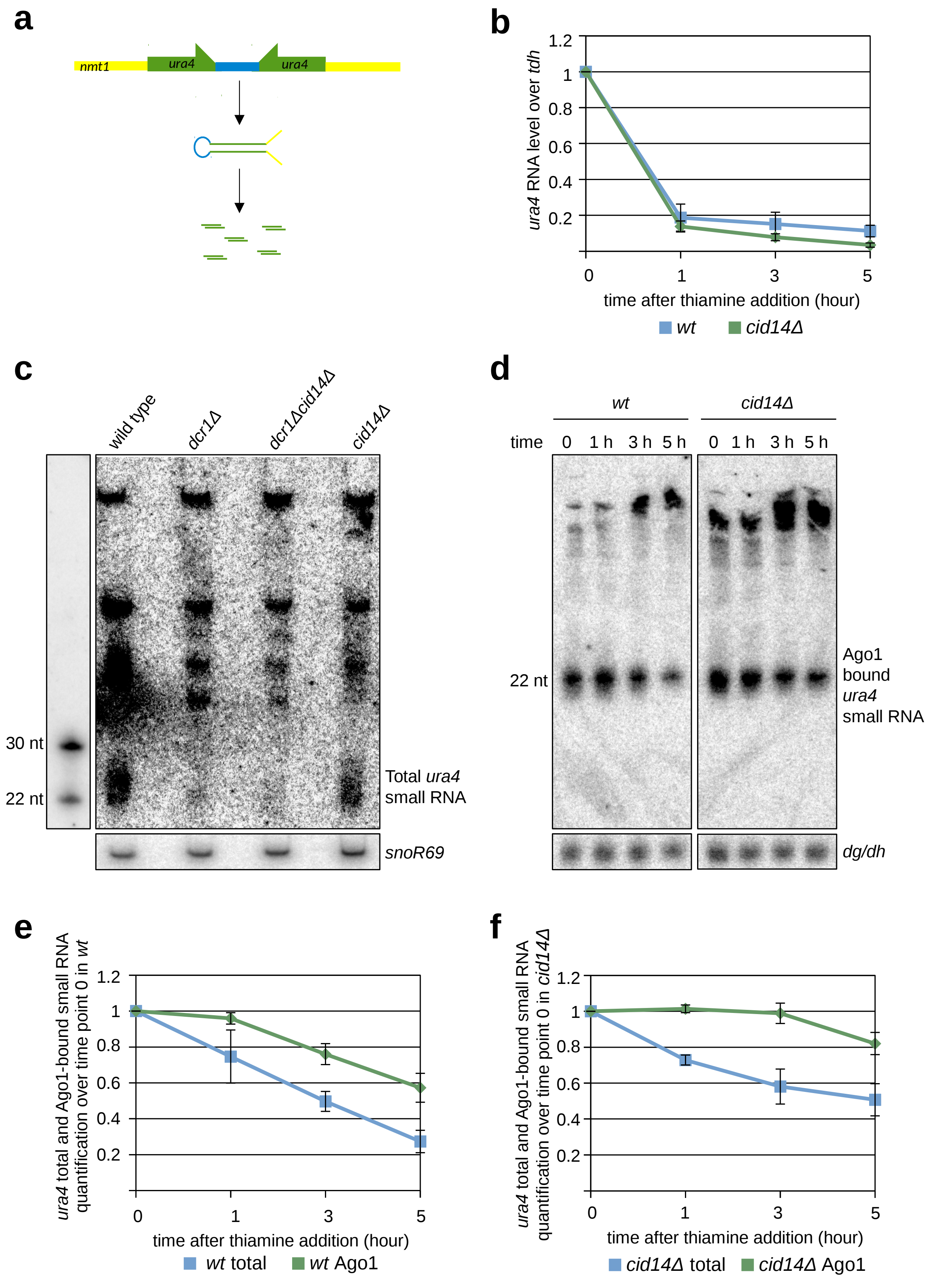

Supplementary Figure 6

Supplementary Figure 6. Argonaute-bound small RNAs have longer half-life in *cid14Δ* cells

(a) Schematic diagram of the *ura4* hairpin construct under the *nmt1* promoter.

(b) Quantification of *ura4* hairpin RNA in indicated strains by RT-qPCR. In *wt* and *cid14Δ* cells *ura4* hairpin mRNA is strongly reduced after 1 hour upon thiamine addition. Error bars indicate standard error s.e.m. of three independent experiments.

(c) Uncropped northern blot showing *ura4* small RNAs isolated from total small RNA fraction from the indicated strains. Lower panel shows the loading control *snoR69*.

(d) Uncropped northern blot showing Argonaute-bound *ura4* small RNAs isolated from wild type and *cid14Δ* cells. In *cid14Δ* cells *ura4* small RNAs have longer half-life than in the wild type cells. Argonaute-bound *ura4* small RNAs were normalized to centromeric small RNAs shown in the lower panel. Quantification is relative to time point 0 when thiamine was added and is shown below the image.

(e) Quantification of total and Argonaute-bound *ura4* small RNAs half-life in *wt*. Quantification is relative to time point 0 after addition of thiamine. Error bars indicate standard error s.e.m. of three independent experiments.

(f) Quantification of total and Argonaute-bound *ura4* small RNAs half-life in *cid14Δ*. Quantification is relative to time point 0 after addition of thiamine. Error bars indicate s.e.m. of three independent experiments.

Supplementary Table 1: Strains used in this study

797	h90 *otr1R*(SphI)::*ura4 + ura4-DS/E leu1-32 ade6-M210*	
63	h+ *otrR*(SphI)::*ura4 ura4 DS/E leu1-32 ade6-M210*	SPY137
65	h90 *otr1R*(SphI)::*ura4 + ura4-DS/E leu1-32 ade6-M210* natMX6::3xFLAG-*ago1*	SPY797
657	h90 *otr1R*(SphI)::*ura4 + ura4-DS/E leu1-32 ade6-M210* kanMX6::3xFLAG-*ago1*	SPY797
80	h+ *leu1-32 ade6-210 ura4DS/E otrR*(SPhI)::*ura4+ clr4Δ*::kanMX6	SPY815
34	h90 *otr1R*(SphI)::*ura4 + ura4-DS/E leu1-32 ade6-M210* natMX6::3xFLAG-ago1 dcr1Δ::hphMX6	
237	SPG1011 h+ leu1-32 ura4-D18 imr1R(NCol)::ura4+ oriI ade6-216 *cid14*Δ::natMX6	SPY787
678	h90 *otr1R*(SphI)::*ura4 + ura4-DS/E leu1-32 ade6-M210* kanMX6::3xFLAG-*ago1 cid16*Δ::natMX6	
708	h90 *otr1R*(SphI)::*ura4 + ura4-DS/E leu1-32 ade6-M210* kanMX6::3xFLAG-*ago1 cid14*Δ::natMX6	
724	h90 *otr1R*(SphI)::*ura4 + ura4-DS/E leu1-32 ade6-M210* kanMX6::3xFLAG-*ago1 cid14*Δ::natMX6 *cid16*Δ::hphMX6	
660	h90 *otr1R*(SphI)::*ura4 + ura4-DS/E leu1-32 ade6-M210* kanMX6::3xFLAG-*ago1 cid12*Δ::natMX6	
677	h90 *otr1R*(SphI)::*ura4 + ura4-DS/E leu1-32 ade6-M210* kanMX6::3xFLAG-*ago1 cid13*Δ::natMX6	
679	h90 *otr1R*(SphI)::*ura4 + ura4-DS/E leu1-32 ade6-M210* kanMX6::3xFLAG-*ago1 cid1*Δ::natMX6	
698	h90 *otr1R*(SphI)::*ura4 + ura4-DS/E leu1-32 ade6-M210* kanMX6::3xFLAG-*ago1 cid12*Δ::natMX6 *cid16*Δ::hphMX6	
703	h90 *otr1R*(SphI)::*ura4+ ura4-DS/E leu1-32 ade6-M210* kanMX6::3xFLAG-*ago1 cid13*Δ::natMX6 *cid16*Δ::hphMX6	
704	h90 *otr1R*(SphI)::*ura4 + ura4-DS/E leu1-32 ade6-M210* kanMX6::3xFLAG-*ago1 cid1*Δ::natMX6 *cid16*Δ::hphMX6	
715	h90 *otr1R*(SphI)::*ura4 + ura4-DS/E leu1-32 ade6-M210* kanMX6::3xFLAG-*ago1 cid11*Δ::natMX6	
720	h90 *otr1R*(SphI)::*ura4 + ura4-DS/E leu1-32 ade6-M210* kanMX6::3xFLAG-*ago1 cid11*Δ::natMX6 *cid16*Δ::hphMX6	
723	h90 *otr1R*(SphI)::*ura4 + ura4-DS/E leu1-32 ade6-M210* kanMX6::3xFLAG-*ago1 cid14*Δ::natMX6 *cid1*Δ::hphMX6	
725	h90 *otr1R*(SphI)::*ura4 + ura4-DS/E leu1-32 ade6-M210* kanMX6::3xFLAG-*ago1 cid14*Δ::natMX6 *cid11*Δ::hphMX6	
744	h90 *otr1R*(SphI)::*ura4+ ura4-DS/E leu1-32 ade6-M210* kanMX6::3xFLAG-*ago1 cid14*Δ::natMX6 *cid13*Δ::hphMX6	

745	h90 *otr1R*(SphI)::*ura4* + *ura4-DS/E leu1-32 ade6-M210* kanMX6::3xFLAG-*ago1* *cid14*Δ::natMX6 *cid12*Δ::hphMX6	
852	h90 *otr1R*(SphI)::*ura4* + *ura4-DS/E leu1-32 ade6-M210* natMX6::3xFLAG-*ago1* kanMX6::3xHA-*cid14*	
941	h90 *otr1R*(SphI)::*ura4* + *ura4-DS/E leu1-32 ade6-M210* kanMX6::3xHA-*cid14*	
650	h90 *otr1R*(SphI)::*ura4* + *ura4-DS/E leu1-32 ade6-M210* natMX6::3xFLAG-*ago1* *dis3l2Δ*:: kanMX6	
11	fWP5 h+ leu1-32 *dis3-54::hphMX6* natMX6::3xFLAG-*ago1*	

Supplementary Table 2: Strains + plasmid

137 + p829	h90 *otr1R*(SphI)::*ura4* + *ura4-DS/E leu1-32 ade6-M210* + pREP1	
65 + p936	h90 *otr1R*(SphI)::*ura4* + *ura4-DS/E leu1-32 ade6-M210* + pREP1-*rdp1*	
708 + p178	h90 *otr1R*(SphI)::*ura4* + *ura4-DS/E leu1-32 ade6-M210* kanMX6::3xFLAG-*ago1* *cid14*Δ*::*natMX6 + *pREP1*	19
708 + p936_2	h90 *otr1R*(SphI)::*ura4* + *ura4-DS/E leu1-32 ade6-M210* kanMX6::3xFLAG-*ago1* *cid14*Δ::natMX6 + pREP1-*rdp1*	26
708 + p936_3	h90 *otr1R*(SphI)::*ura4* + *ura4-DS/E leu1-32 ade6-M210* kanMX6::3xFLAG-*ago1* *cid14*Δ::natMX6 + pREP1-*rdp1*	24
657 + p895	h90 *otr1R*(SphI)::*ura4*+ *ura4-DS/E leu1-32 ade6-M210* natMX6::3xFLAG-*ago1* + pREP1-*ura4sh5*	55/56
708 + p895	h90 *otr1R*(SphI)::*ura4*+ *ura4-DS/E leu1-32 ade6-M210* kanMX6::3xFLAG-*ago1* *cid14*Δ::natMX6 + pREP1-*ura4sh5*	57/58
34 + p895	h90 *otr1R*(SphI)::*ura4* + *ura4-DS/E leu1-32 ade6-M210* natMX6::3xFLAG-ago1 dcr1Δ::hphMX6 + pREP1-*ura4sh5*	62/63
785 + p895	dcr1Δ::hphMX6 *cid14*Δ::natMX6 + pREP1-*ura4sh5*	65

Supplementary Table 3: Plasmids used in this study

85	pFA6a + 3xHA-kan	p434
776	pRSF_Duet_GST_PB + Cid14-6His	
779	pREP1_nmt1_FLAG + Cid16-GlySerSer-6His	
218	pREP1_nmt1_FLAG + Rdp1	p936
860	pET-28b + Rrp6-Flag	pFB296
895	pREP1_nmt1 + *ura4sh5*	
813	pREP1_nmt1_FLAG + Cid16DADA-GlySerSer-6His	
814	pRSF_Duet_GST_PB + Cid14DADA-6His	

Supplementary Table 4: Oligonucleotides used in this study

110a F	tdh1	CCAAGCCTACCAACTACGA	
110a R	tdh1	AGAGACGAGCTTGACGAA	RT
110f F	cen dg	CTGCGGTTCACCCTTAACAT	
110f R	cen dg	CAACTGCGGATGGAAAAAGT	RT
264 F	rdp1	TCCTGCTATGTGCTCTGGTG	
264 R	rdp1	CCAAATATCCCTTCCGGATT	RT
1006 F	ura4-hp5	CTTTAAGCAAGAGAATCATATGTGTCGA	RT
1006 R	ura4-hp5	CTTACCGTTTTGGAGATCCCG	RT
466	snoR69	CAATGTAAATACTCCGAGTGAGCTGGGTTTAAC	NB
467 a	cen dg	ATTTGACGAGGCACATTCCTTA	NB
467 b	cen dg	AATTTGACGAGGCACATTCCTTA	NB
467 c	cen dh	CAGGAGTTGCGCAAACGAAGTTA	NB
467 d	cen dg	ACCGAGTGCAAATGCTTTTGTA	NB
467 e	cen dg	CTGACTTGGCTTGTCTTCTGTA	NB
467 f	cen dg	GGCATAGCGATGATAGTTCTA	NB
467 g	cen dg	AGGCATAGCGATGATAGTTCTA	NB
467 h	cen dh	GACGATAAGCAGGAGTTGCGCA	NB
468 a	cen dh	GAGCATTGTAAAATCATTGCTGA	NB
468 c	cen dh	TGCTGAGTTAATTTTTAGTGGA	NB
468 g	cen dh	CAAAGCTTTCAATTTTTTTAGTA	NB
469 a	cen dh	ACTCATTTATTGAATCTGGTGA	NB
469 b	cen dh	GCATCAAAGGAATGTTTCCTCA	NB
469 c	cen dh	CAATCTTTAAATTCCTTTCTGA	NB
946 a	ura4 siRNA	AGTTGGTTTACCTTTGGGACGTGGTCTCTTGCTTTTGGCT	NB
255	ss 22 nt RNA	UGAAAGCUUUAGUUGAUACGUC	assay
71	ds 22 nt RNA	GCGAGCGAGGCAAAGAACAAGA	assay
72	ds 22 nt RNA	UUGUUCUUUGCCUCGCUCGCUG	assay
461 F	cid14	GACTGGATCCATGGGTAAAAAAAGCGTG	cloning
461 R	cid14	GACTGATATCCTAAAAACGTTTGCGTATTTTTTTC	cloning
460 F	cid16	GACTGGCGCGCCTATGCTATTTGCCAAATTATTG	cloning
665 R	cid16	GACTCCCGGGTTATTGAATCAAGGGATCCAG	cloning

712 F	cid14	CACCATCATCACCAC-TAGGATATCGGCC	iPCR
712 R	cid14	ATGGCTGCTGCC-AAAACGTTTGCGTATTT	iPCR
713 F	cid16	CACCATCATCACCAC-TAACCCGGGGCG	iPCR
713 R	cid16	ATGGCTGCTGCC-TTGAATCAAGGGATCC	iPCR
523 AF	cid14	CGGAAGACGAAGTACCTATTATTGAGGACACCACTGCTTCAGATGAAGAATCTCGAGCGAAAAAAATACGCAAACGTTTTCGGATCCCCGGGTTAATTAA	ET
523 R	cid14	ACAATTATCAAACTATAATCATAATTGATAATAAAACCTTTTATCCCTCTATATAACCTGGTATTTTACATGTAAATTAAGAATTCGAGCTCGTTTAAC	ET
524 AF	cid16	GCTATGAACTCGAAAGAGCGTGTAGAATTTTAAGCGATCCAAAATGTAATCTAGATCATTTACTGGATCCCTTGATTCAACGGATCCCCGGGTTAATTAA	ET
524 R	cid16	ACCGGCCAACGGTATTTTGAAAGTGAGTCAGAGAGGGAAAAAACTGTTTTTTTCTGTTCTTATGTTCATATATAAAAGATGAATTCGAGCTCGTTTAAAC	ET

Primers used for ChIP and qRT-PCR if no specification, RT: reverse transcription, NB: Northern Blot, ET: endogenous tagging, iPCR: inverse PCR, underlined sequences: restriction sites.

Supplementary Table 5: Sequencing data

**small RNA sequencing :**	**small RNA sequencing :**
wild type (797-2) (GEO: GSE19734)	*cid14Δcid13Δ* (744)
wild type (797-20) (GEO: GSE19734)	*cid14Δcid12Δ* (745)
wild type (657)	*cid16Δcid12Δ* (698)
cid14Δ (708)	*cid16Δcid13Δ* (703)
cid16Δ (678)	*cid16Δcid1Δ* (704)
cid12Δ (660)	*cid16Δcid11Δ* (720)
cid13Δ (677)	*dcr1Δ* (34) (GEO: GSE38636)
cid1Δ (679)	*rrp6Δ* (504) (GEO: GSE38636)
cid11Δ (715)	*tri1Δ* (136) (GEO: GSE38636)
cid14Δcid1Δ (723)	*rrp6Δtri1Δ* (549) (GEO: GSE38636)
cid14Δcid16Δ (724)	*dis3l2Δ* (650)
cid14Δcid11Δ (725)	*cid14Δ* + pREP1-Rdp1_2 (26)
dis3-54 (11) (GEO: GSE19734)	*cid14Δ* + pREP1-Rdp1_3 (24)
**RNA sequencing :**	**ChIP sequencing :**
wild type (657)	*wild type* + pREP1 (137+p829)
cid14Δ (708)	*cid14Δ* + pREP1 (19)
cid16Δ (678)	*cid14Δ* + pREP1-Rdp1_2 (26)
dcr1Δ (34)	
dcr1Δ (34) (GEO: GSE38636)	

Supplementary References:

1. Bühler, M., Haas, W., Gygi, S. P. & Moazed, D. RNAi-dependent and -independent RNA turnover mechanisms contribute to heterochromatic gene silencing. *Cell* **129,** 707–721 (2007).
